# Expression of CD64 and CD69 as biomarkers for late-onset sepsis diagnosis in infants born prematurely

**DOI:** 10.1016/j.bjid.2025.104511

**Published:** 2025-02-19

**Authors:** Alicia Ramírez-Ramírez, Ismael Mancilla-Herrera, Ricardo Figueroa-Damián, Diana Soriano-Becerril, Graciela Villeda-Gabriel

**Affiliations:** Instituto Nacional de Perinatología, Departamento de Infectología e Inmunología, Ciudad de México, México, USA

**Keywords:** Early immune response, Neonatal sepsis diagnosis, Surface leukocyte markers

## Abstract

**Background:**

The incidence of Late-Onset Sepsis (LOS) increases as gestational age decreases in newborns. The clinical signs of neonatal sepsis are not specific for diagnosis in preterm infants. The gold standard for its diagnosis is the blood culture test, which requires more than 24 h to obtain results, with positive results obtained in 10–3 % of cases analysed. As the molecular markers on the lymphocyte surface CD64 and CD69 are involved in early innate immune activation, they may be helpful for faster diagnosis.

**Aim:**

Measure the expression of CD64 and CD69 on lymphocytes in clinical and confirmed sepsis patients and compared to that in infants without sepsis.

**Methodology:**

We used peripheral blood samples from three groups of preterm babies with suspected sepsis (*n* = 31), confirmed sepsis (*n* = 10) and without sepsis (*n* = 47). Using flow cytometry, we measure the expression of CD64 on neutrophils and CD69 on NK cells.

**Results:**

Expression of CD64 on neutrophils and CD69 on NK cells did not increase in the clinical or confirmed sepsis groups compared to the without sepsis group.

**Conclusions:**

Leukocytes from infants born prematurely may have tightly regulated mechanisms that control their activation phenotype, rendering them unsuitable for diagnosing sepsis.

## □

Sepsis has been defined as a clinical syndrome that denotes a life-threatening state of organ dysfunction triggered by an imbalanced host response to an infection, as outlined in the Sepsis-3 criteria. Infections giving rise to sepsis may stem from various sources, including bacterial, fungal, parasitic, or viral origins.^1^^,^^2^ A recent meta-analysis approximates the global prevalence of neonatal sepsis at 937 cases per 100,000 live births.^3^ A recent systematic review and meta-analysis reported that the Early-Onset Sepsis (EOS) incidence is 2496 per 100,000 live births, which was 2.6 times more common than Late-Onset Sepsis (LOS), 946 per 100 live births. The incidence of EOS has been declining throughout the years, from 1990 to 2015, from 1.37 to 0.23 per 1000 live births. This is because of universal group B streptococcus screening and intrapartum antibiotic prophylaxis. Nonetheless, the incidence of LOS has remained nearly unchanged, at 0.31 per 1000 live births,^4^ however morbidity and mortality following neonatal sepsis is greatest in low- and middle-income countries where data is scarce.^5^ The prevalence of sepsis is significantly higher in both preterm and low-weight new-borns, with a reported mortality of 17.6 %.^4^

Frequently, neonatal sepsis progresses with complications that lead to prolonged hospitalisation because patients can develop one or more infections.^6^ The identification of neonatal sepsis at its onset poses a challenge as it primarily depends on non-specific and inconsistent clinical indicators. Although blood culture is the gold standard method for neonatal sepsis diagnosis, the lengthy turnaround time^7^ (up to 24 hours) and several factors, including intermittent or low bacteraemia, maternal intrapartum antimicrobial exposure, and small blood volumes obtained from preterm babies, make it a low-sensitivity assessment method.^8^^,^^9^ Consequently, antibiotics are often administered to suspected neonates before blood culture results are available to prevent rapid clinical deterioration despite the risk of unnecessary antibiotic exposure.^6^^,^^10^ This overexposure to antibiotics is concerning, as it can adversely affect the gut microbiota and contribute to the development of antimicrobial resistance, potentially leading to necrotising enterocolitis, premature death, the development of asthma, allergies, autism spectrum disorders, and metabolic disorders later in life. To prevent the unnecessary administration of antibiotics to uninfected individuals, developing a highly sensitive biomarker with a substantial negative predictive value is imperative.^10^^,^^11^ Infection drives immune cell activation by increasing expression of cell surface receptors and cell surface markers, including CD64 on neutrophils and CD69 on NK cells, which have been indicated as potential biomarkers for early diagnosis of neonatal sepsis.^12^, ^13^, ^14^, ^15^

This study examined CD64 expression on neutrophils and CD69 expression on NK cells by flow cytometry in babies who were born preterm (< 37-weeks of gestation) with clinical symptoms of sepsis. This was a prospective study of infants born prematurely and cared for at the Instituto Nacional de Perinatología Isidro Espinosa de los Reyes (INPerIER), Mexico City, with suspected LOS.

In this study, samples from babies older than 7-days with suspected LOS were initially included successively. After we applied a non-probabilistic criterion sampling (samples selection was based on the blood culture result) to form the study groups.

The criteria of the neonatologist [1] for the inclusion of infants were fever or hypothermia, tachycardia or bradycardia, tachypnoea or altered leukocyte numbers, Positive Procalcitonin Test (PCT), and positive C Reactive Protein (CRP) test. Blood samples were taken when signs appeared and the samples for CBC and CRP tests were taken at the same time that the blood culture. These samples were collected in Microtainer MAP Tube with K2EDTA and Microtainer SST amber with silica and separating gel (BD. San José, CA, USA), respectively.

Then the infants were included in the suspected sepsis group. Furthermore, babies without signs of sepsis and those who required CRP and Complete Blood Count (CBC) testing due to a medical monitoring process for having exhibited a condition other than sepsis, were included in the control group. The most frequent condition in these infants were Transient Tachypnoea of the Newborn (TTN). TTN is not a serious process and may be temporary (it usually resolves within 72 hours of birth) and many of these control babies were not hospitalised and went home in two or three days after birth.

We did not include babies with major congenital malformations or those who needed immediate surgery or blood transfusion.

The INPerIER Research, Ethics and Biosecurity Committees approved and funded the study under approval number INPer_2015–1-153. The collection of samples for this study did not involve any additional risk to the patients since the biological samples were taken from the remnant for other clinical tests (CRP and CBC), for which the parents of the newborns signed an informed consent form. Therefore, this study met the bioethics criteria established by the Declaration of Helsinki guidelines.

To determine expression of CD64 and CD69 on neutrophils and NK cells, respectively, fifty microlitres of whole peripheral blood samples from each patient were used for immunostaining. Each sample was incubated with previously titrated monoclonal fluorochrome-conjugated human antibodies (BioLegend, San Diego, CA, USA): anti-CD45 APC/Cy7, anti-CD56 Brilliant Violet, anti-CD14 PE-Cy7, anti-HLA-DR PE-Cy5, anti-CD16 FITC, anti-CD64 APC and anti-CD69 PE. Erythrocytes were lysed, and 10,000 lymphocytes were acquired from each sample using a FACS ARIA III flow cytometer using BD FACSDiva software V.7.0 (BD Biosciences, San Jose, CA, USA). Neutrophils and NK cells were selected, and CD64 and CD69 expression is reported as the Mean Fluorescence Intensity (MFI).

The Jarque-Bera test was performed to determine the normality of the data. Differences between groups were calculated using the Chi-Square test, one-way analysis of variance or the Kruskal-Wallis test and Fisher's exact test. Central tendency measures are expressed as means with a standard deviation or frequency. We used Prism version 7 software (Boston, MA, USA).

We analysed 85 infants born prematurely with suspected sepsis. 10 (11.8 %) babies had a positive blood culture test, confirming neonatal sepsis. The following microorganisms were isolated: *Klebsiella pneumoniae, Staphylococcus epidermidis, Enterococcus* sp., *Kodamaea ohmeri, Serratia marcenses*, and *Candida* sp. The infants with positive blood cultures composed the Confirmed Sepsis group (CS).

From the same group of infants with suspected sepsis and negative blood culture, remained 75 samples. Then, we selected samples from babies who had a similar gestational age to those in the CS group and the Clinical Sepsis group (CLS) were composed with 31 of these samples. The control group was recruited the same way as the suspected sepsis babies were. First, the samples were processed in a consecutive way with the characteristics of inclusion: preterm babies without signs of sepsis but with a CBC and CRP test request due to clinical monitoring. Then, the samples were selected by the gestational age of the babies and the Non-Sepsis group (NS) were composed, in which 47 samples were included. In this group, also the gestational age had to be similar to that of the babies in the CS group. Table 1 summarises the demographic and clinical characteristics of these three groups.

**Table 1:** Demographic and clinical characteristics of the newborn groups.

	NS (*n* = 47)	CLS (*n* = 31)	CS (*n* = 10)	p-value
	Mean ±SD	Mean ±SD	Mean ±SD	
**Sex (F/M)**	17/30	14/17	5/5	0.6034^a^
**Birth weight (g)**	1405 ± 633.5	1334 ± 596.6	1373 ± 818.7	0.8917^b^
**Gestational age (weeks)**	30.66 ± 2.76	30.45 ± 3.38	30.95 ± 3.98	0.8995^c^
**Apgar score (1-min)**	5.63 ± 2.18	5.51 ± 2.12	4.50 ± 1.9	0.2386^c^
**Apgar score (5-min)**	8.21 ± 1.44^d^	7.77 ± 1.74	7.2 ± 1.55^d^	0.0492^c^
**Silverman Anderson score**	5.49 ± 4.09	6.25 ± 4.29	2.0 ± 1.0	0.2195^b^
**Postnatal age (days)**	24.02 ± 20.65	33.65 ± 32.11	34.20 ± 41.12	0.2649^b^
	**n (%)**	**n (%)**	**n (%)**	
**Product of multiple pregnancy**	11 (23)	17 (55)	1 (10)	0.0040^a^
**Maternal infection data**	15 (32)	11 (35.5)	2 (20)	0.4741^a^
**Antenatal steroids**	16 (34)	10 (32)	4 (33)	0.5501^a^
**Caesarean section**	45 (96)	24 (77)	8 (80)	0.1760^a^
**High CRP test values**	8 (17)	18 (58)	8 (80)	0.4528^f^
**High PCT values**	1 (2.13)	8 (26)	5 (50)	0.0289^f^
**Fever**	0	14 (45)	6 (60)	0.4841^f^
**Apnoea**	0	10 (32)	2 (20)	0.4577^f^
**Altered complete blood count**	0	18 (58)	6 (60)	1.000^f^
**Septic shock**	0	2 (6)	1 (10)	1.000^f^

NS, Nonsepsis; CLS, Clinical sepsis; CS, Confirmed sepsis; F, Female; M, Male; SD, Standard deviation; CRP, C Reactive Protein (Positive cutoff:> 6 mg/mL); PCT, Procalcitonin Test (Positive cutoff:≥ 0.5 ng/mL).

a Chi-square test.

b One-way ANOVA test.

c Kruskal Wallis test. d,e Statistically significant with Tukey's post-analysis test.

f Fisher's exact test between CLS and CS group.

**Fig. 1:**
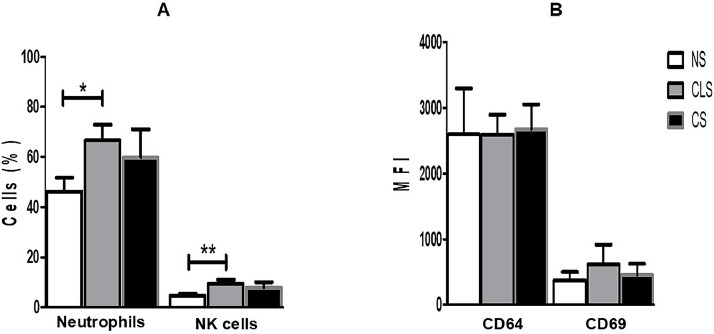
(A) Percentages of neutrophils and NK cells in blood samples from preterm newborns with clinical sepsis (CLS [*n* = 31]) or confirmed sepsis (CS [*n* = 10]) and nonsepsis (NS [*n* = 47]) disease. The percentage of cells was compared with one-way ANOVA and Tukey's multiple comparison post-hoc test. *p < 0.05; **p < 0.01, ***p < 0.001. (B) Expression of CD64 on neutrophils and CD69 on NK cells in blood samples from preterm newborns with clinical sepsis (CLS [*n* = 31]) or confirmed sepsis (CS [*n* = 10]) and nonsepsis (NS [*n* = 47]). Expression of CD64 and CD69 was compared between groups by one-way ANOVA and Tukey's multiple comparison post-hoc test. MFI, Mean Fluorescence Intensity.

Expression of CD64 and CD69 on neutrophils and NK cells, respectively, was assessed by flow cytometry and expressed as Mean Fluorescence Intensity (MFI); the percentages of neutrophils and NK cells were also determined (Fig. 1A). Despite a significant increase in the percentage of neutrophils and NK cells in the CLS group, we did not find significantly elevated expression of CD64 or CD69 on these cells from the CLS or CS group compared to the control group. In contrast, we detected basal expression of CD64 and CD69 in the control group (NS), which was also present in the CLS and CS groups in amounts similar to those in the control group (Fig. 1B).

CD64 (FcγRI) is a receptor that binds to the Fc portion of gamma immunoglobulin; is present in macrophages, dendritic cells, and the surface of monocytes; and plays an essential role in innate immunity by promoting neutrophilic phagocytosis. Under physiological conditions, neutrophils express low levels of CD64. These levels increase four to six hours after onset of a bacterial infection or inflammatory damage and return to baseline levels when the stimulus disappears.^2^ Conversely, CD69 is a biomarker of early activation of lymphocytes and NK cells. When upregulation occurs within 2 hours after stimulation, it reaches its highest level at 24 hours and can still be detected after 75 hours.^11^^,^^16^ CD69 expression by NK cells is induced by infection with different microorganisms, interferons, and tumour necrosis factor alpha, which promote release of proinflammatory cytokines.^6^^,^^10^

The significantly increased level of CD64 expression in neutrophil and increased expression of CD69 on NK cells in samples from infants with confirmed infection compared to controls without sepsis has been reported as a potential biomarker for early diagnosis of neonatal sepsis.^12^, ^13^, ^14^ Our results revealed that CD64 and CD69 expression did not increase significantly in the CLS or CS group compared to the NS group (Fig. 1B), which is contrary to the findings of previous studies but is consistent with those reported by Yang et al. (2016) and Cérbulo-Vazquez et al. (2003), who confirmed no elevated expression of CD64 on neutrophils or CD69 on NK cells from preterm sepsis patients.^15^^,^^17^

In our population, the lack of activation of innate immune cells might be due to the regulated production of proinflammatory cytokines in preterm infants.^18^^,^^19^ The reason for this is that in newborns, foetomaternal tolerance must be preserved during pregnancy, the transition from a sterile intrauterine environment to a nonsterile external environment must be allowed for a microbial colonisation after birth must be established. This response promotes a regulatory Th2/Th17-type response instead of a Th1-type or proinflammatory response. However, this mechanism might have severe consequences in preterm infants, increasing their susceptibility to microbial infections,^18^^,^^19^ and making CD64 and CD69 expression unsuitable biomarkers for diagnosis of neonatal sepsis.

Although some authors have concluded that expression of CD64 and CD69 is not affected by the gestational age of the patient, physicians should consider expression of leukocyte surface markers for diagnosis in preterm infants with caution.

Overall, development of suitable and novel biomarkers involves an in-depth study and characterisation of the innate immune response in a broad spectrum of clinical cases, populations, and other relevant characteristics present in preterm and term infants.

## Conflicts of interest

The authors declare no conflicts of interest.
